# Alteration of gene expression in mice after glaucoma filtration surgery

**DOI:** 10.1038/s41598-020-72036-0

**Published:** 2020-09-14

**Authors:** Keisuke Adachi, Yosuke Asada, Toshiaki Hirakata, Miki Onoue, Satoshi Iwamoto, Toshimitsu Kasuga, Akira Matsuda

**Affiliations:** Laboratory of Ocular Atopic Diseases, Department of Ophthalmology, Juntendo University School of Medicine, 2-1-1 Hongo, Bunkyo-Ku, Tokyo, 113-8431 Japan

**Keywords:** Eye diseases, Acute inflammation

## Abstract

To clarify the early alterations of gene expression using a mouse model of glaucoma filtration surgery, we carried out microarray expression analysis. Using BALB/c mice, a filtration surgery model was made by incision of the limbal conjunctiva, followed by the insertion of a 33G needle tip into the anterior chamber, and 11-0 nylon sutures. Subgroups of mice were treated intraoperatively with 0.4 mg/ml mitomycin-C (MMC). At day 3 after surgery the bleb was maintained. The bleb region tissue was sampled 3 days after the filtration surgery, and gene expression analysis was carried out using a mouse Agilent 8 × 60 K array. We found 755 hyperexpressed transcripts in the bleb region compared to control conjunctiva. The hyperexpressed transcripts included epithelial cell metaplasia-related (*Il1b*, *Krt16*,* Sprr1b*), inflammation-related (*Ccl2*,* Il6*) and wound healing-related (*Lox*,* Timp1*) genes. We also found downregulation of a goblet cell marker gene (*Gp2*) in the bleb conjunctiva. MMC treatment suppressed elastin (*Eln*) gene expression and enhanced keratinization-related gene expression (*Krt1*,* Lor*) in the bleb region. Our results suggest the importance of epithelial wound healing after filtration surgery, and this filtration surgery model will be a useful tool for further pathophysiological analysis.

## Introduction

Glaucoma is an optic neuropathy characterized by degeneration of retinal ganglion cells and cupping of the optic nerve head as well as visual field defects^1,2^. Reduction of intraocular pressure is the method of treatment for glaucoma. Although anti-glaucoma medication and/or restoration of the aqueous humor drainage pathway by laser treatment or by minimally invasive glaucoma surgery are first-line of the treatments in most of the cases, glaucoma filtration surgery is required in severe cases because of its high effectiveness in reducing the pressure. However, this surgery has a higher risk of complications like bleb leaks, bleb infection and hypotony. Moreover, fibrotic changes in the bleb region after filtration surgery induce re-elevation of intraocular pressure^3^. Previous reports using rabbit^4^ and rat models^5^ of filtration surgery showed activation of fibrosis-related genes. In human studies, increased levels of TGF-β2 in the aqueous humor^6^, vascular endothelial growth factor (VEGF) in tenon tissue^7^, and lysyl oxidase-like 2 (LOXL2) in the aqueous humor and tenon tissue^8^ have been reported in relation to scarring of the filtration bleb. To further investigate the mechanism of bleb fibrosis, we established a mouse model of filtration surgery and analyzed gene expression profiles using a microarray. Mouse models have several advantages over rabbit and rat models because of (1) the availability of research tools like gene arrays, antibodies and recombinant proteins, and (2) the availability of gene-modified mouse models for functional studies. We established a mouse model of filtration surgery using 33G needle tips, which enabled aqueous humor filtration and bleb formation, and carried out microarray analysis to elucidate genes that were differentially expressed (DEGs) in the bleb region and control conjunctiva. The final goal of our glaucoma model study is to find some clues to improve the clinical outcome of glaucoma filtration surgery. In this study, we tried to clarify the early alterations of gene expression in a mouse model of glaucoma filtration surgery, using microarray expression analysis as an initial step. We also examined the effect of mitomycin-C (MMC), which is widely used clinically as a wound healing process modifier and to enhance the bleb survival rate in glaucoma filtration surgery^9^.

## Results

### Microarray analyses revealed differentially expressed transcripts in a mouse glaucoma filtration surgery model

**Figure 1:**
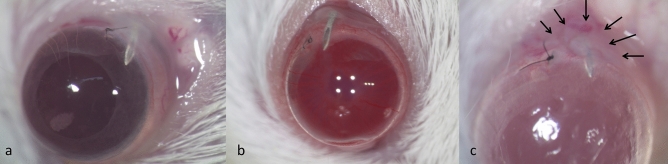
Photographs of mouse eyes after filtration surgery. Photograph of mouse eyes one day (**a**), three days (**b**) and seven days (**c**) after filtration surgery. At day 1 and day 3, the bleb was maintained, but at day 7 the bleb region was encapsulated (arrows).

From one day after surgery bleb formation was observed (Fig. 1a) and the bleb was maintained at day 3 after surgery (Fig. 1b). At 7 days after surgery, the bleb region became largely encapsulated (Fig. 1c, arrows). By applying gentle pressure on the cornea the bleb was reformed in some cases (Supplementary Fig. S2).

Microarray analyses were carried out for three conditions; (1) the filtration bleb, (2) the filtration bleb with MMC and (3) control tissue. We found 755 transcripts hyperexpressed in day 3 filtration samples (Supplementary Table S1) and the top 39 hyperexpressed transcripts are shown in Table 1. We carried out gene ontology (GO) analysis and found 124 GO pathways enriched in the 39 hyperexpressed transcripts (Supplementary Table S5) and selected 6 representative GO pathways (Supplementary Table S6) by removing redundant pathways. We found hyperexpression of genes related to responses to external stimuli (*Il1b*, *Krt16*,* Sprr1b*), inflammation-related genes (*Ccl2*,* Cxcl1*,* Cxcl5*,* Il6*,* Saa1*,* S100a8*), and wound healing-related genes (*Lox*,* Timp1*) after filtration surgery.

**Table 1:** Top 39 hyperexpressed transcripts (filtration > control).

q-value^a^	Fold change [Filt]/[control]	Ave filtration (normalized)	Ave Control (normalized)	Gene Symbol
0.0012	2,653.93	3.53	− 7.85	*Sprr1b*
0.0026	1,235.65	6.63	− 3.64	*Saa3*
0.0013	391.45	5.78	− 2.83	*Ppbp*
0.0016	366.91	1.74	− 6.78	*Sprr1b*
0.0030	211.00	1.67	− 6.06	*Il6*
0.0019	207.43	3.31	− 4.39	*Clec4d*
0.0019	200.87	1.73	− 5.92	*Acod1*
0.0078	182.77	2.59	− 4.92	*Sprr2a3*
0.0047	152.55	2.52	− 4.73	*Serpinb2*
0.0422	127.29	2.85	− 4.14	*Sprr2f*
0.0013	117.62	6.75	− 0.13	*S100a9*
0.0046	110.05	3.38	− 3.41	*Lrrc15*
0.0014	109.07	4.61	− 2.16	*Cxcl5*
0.0016	102.73	5.09	− 1.59	*Krt16*
0.0015	100.93	6.09	− 0.57	*Krt16*
0.0020	100.63	4.68	− 1.97	*S100a8*
0.0023	73.14	1.56	− 4.63	*Mefv*
0.0011	68.24	6.72	0.62	*Timp1*
0.0017	50.72	3.44	− 2.23	*Il1b*
0.0028	40.47	1.99	− 3.35	*Adamts4*
0.0031	31.22	2.29	− 2.68	*Sirpb1b*
0.0024	30.20	3.02	− 1.90	*Cnfn*
0.0039	30.20	1.92	− 3.00	*Apoc2*
0.0012	25.91	3.38	− 1.32	*Saa1*
0.0222	23.68	3.89	− 0.68	*Hp*
0.0014	21.80	4.54	0.09	*Lox*
0.0013	21.25	2.48	− 1.93	*Hdc*
0.0047	20.57	1.64	− 2.72	*Stfa2*
0.0019	20.13	2.56	− 1.77	*Fpr2*
0.0011	20.11	1.97	− 2.36	*Lox*
0.0022	19.93	3.07	− 1.25	*Clec4n*
0.0017	18.73	2.89	− 1.34	*Cxcl1*
0.0082	18.57	1.81	− 2.40	*Pira2*
0.0013	18.30	3.68	− 0.51	*Ccl2*
0.0025	18.29	4.25	0.05	*Prg4*
0.0067	18.05	2.25	− 1.93	*Tnfaip6*
0.0029	17.92	2.39	− 1.77	*Ms4a6d*
0.0045	17.07	4.97	0.87	*Lilrb4a*
0.0019	17.00	2.70	− 1.39	*Ccl7*

^a^Filtered by the false discovery rate (*q* < *0.05*): unpaired t-test with Benjamini–Hochberg multiple testing correction. Ave Filtration: normalized average expression value of the day 3 filtration bleb sample. Ave control: normalized average expression value of the control sample.

We found 71 hypoexpressed transcripts in day 3 filtration samples (Supplementary Table S2) and sorted them in the order of fold changes. Among the top 21 hypoexpressed transcripts (Table 2), we found *Gp2* (molecular marker for goblet cells), *Gsta3* (glutathione S-transferase), and *Adh1* genes (Table 2).

**Table 2:** Top 21 hypoexpressed transcripts (filtration < control).

q-value^a^	Fold change [Filt]/[control]	Ave filtration (normalized)	Ave control (normalized)	Gene Symbol
0.007	0.05	0.33	4.56	*Retnla*
0.040	0.12	3.25	6.26	*Lypd2*
0.020	0.18	3.04	5.54	*Gp2*
0.012	0.19	3.12	5.48	*Adh1*
0.019	0.21	4.59	6.85	*Alox12e*
0.026	0.24	3.08	5.16	*Dapl1*
0.027	0.24	3.56	5.62	*Pvalb*
0.007	0.26	3.72	5.64	*Gsta3*
0.032	0.28	2.68	4.52	*Reep6*
0.012	0.28	1.76	3.57	*Inmt*
0.008	0.28	2.98	4.80	*Gsta3*
0.012	0.30	2.56	4.31	*Aldh1a1*
0.017	0.31	3.56	5.27	*Ltbp4*
0.019	0.31	4.40	6.10	*Cbr2*
0.018	0.31	2.45	4.12	*Cyp4a12b*
0.033	0.31	4.51	6.17	*Timp3*
0.010	0.32	3.16	4.82	*Gstm1*
0.012	0.32	4.82	6.47	*Epas1*
0.015	0.32	1.94	3.59	*Cyp4a12a*
0.029	0.32	3.65	5.29	*Timp3*
0.012	0.32	2.97	4.61	*Gstm3*

^a^Filtered by the false discovery rate (*q* < *0.05*): unpaired t-test with Benjamini–Hochberg multiple testing correction. Ave Filtration: normalized average expression value of the day 3 filtration bleb sample. Ave control: normalized average expression value of the control sample.

Most of the upregulated genes (Table 3) in the MMC-treated samples were keratinization-related genes (*Lec1d*,* Krt1*,* Lor[Loricrin]*). The top 18 MMC-repressed transcripts included muscle tissue-related genes, *Eln* (elastin) *Myh3*, and *Myl4* (Table 4).

**Table 3:** Top 21 MMC-induced transcripts.

q-value^a^	Regulation	Fold change [MMC]/[filt]	Ave filtlation	Ave MMC	Gene Symbol
0.007	Up with MMC	324.34	− 3.81	4.53	*Lce1d*
0.007	Up with MMC	191.49	− 3.65	3.93	*Lce1e*
0.013	Up with MMC	151.93	− 3.84	3.41	*Lce1a1*
0.013	Up with MMC	123.60	− 3.54	3.41	*Lce1f*
0.007	Up with MMC	116.90	− 3.23	3.64	*Lce1i*
0.045	Up with MMC	114.64	− 4.96	1.88	*Lce3e*
0.043	Up with MMC	95.34	− 2.95	3.62	*Flg*
0.013	Up with MMC	89.82	− 3.36	3.13	*Lce1b*
0.023	Up with MMC	55.67	− 3.32	2.48	*Lor*
0.038	Up with MMC	54.08	− 3.83	1.92	*Rptn*
0.013	Up with MMC	50.73	− 2.80	2.87	*Lce1g*
0.018	Up with MMC	45.94	− 2.63	2.89	*Hrnr*
0.007	Up with MMC	39.83	− 2.55	2.77	*Nkx2-9*
0.046	Up with MMC	34.38	− 3.46	1.65	*Klk14*
0.013	Up with MMC	34.11	− 2.23	2.86	*Lce1h*
0.038	Up with MMC	33.50	− 2.66	2.41	*Sprr2i*
0.013	Up with MMC	33.35	− 2.10	2.96	*Serpinb12*
0.023	Up with MMC	32.85	− 2.92	2.12	*Krt1*
0.020	Up with MMC	30.41	− 2.25	2.67	*Defb3*
0.023	Up with MMC	29.83	− 2.32	2.58	*Lce1m*
0.040	Up with MMC	26.18	− 3.29	1.42	*Lce3f*

^a^Filtered by the false discovery rate (*q* < *0.05*): unpaired t-test with Benjamini–Hochberg multiple testing correction. Ave Filtration: normalized average expression value of the day 3 filtration bleb sample. Ave MMC: normalized average expression value of the day 3 filtration bleb sample treated with MMC.

**Table 4:** Top 18 MMC-repressed transcripts.

q-value^a^	Regulation	Fold change [MMC]/[filt]	Ave Filt	Ave MMC	Gene Symbol
0.037	Down with MMC	0.07	1.58	− 2.33	*Esp6*
0.040	Down with MMC	0.08	5.15	1.57	*Cyp2a4*
0.040	Down with MMC	0.10	5.90	2.56	*Cyp2a5*
0.040	Down with MMC	0.18	1.94	− 0.51	*Cyp4a12a*
0.045	Down with MMC	0.20	2.45	0.15	*Cyp4a12b*
0.040	Down with MMC	0.23	4.21	2.10	*Myl4*
0.039	Down with MMC	0.24	2.00	− 0.08	*Myl4*
0.042	Down with MMC	0.25	2.18	0.17	*Tnni1*
0.044	Down with MMC	0.25	4.73	2.75	*Myh3*
0.037	Down with MMC	0.27	1.02	− 0.88	*Tnnt2*
0.040	Down with MMC	0.32	1.00	− 0.65	*Myom2*
0.039	Down with MMC	0.34	3.90	2.33	*Cdkn1c*
0.037	Down with MMC	0.35	1.55	0.03	*Eln*
0.039	Down with MMC	0.35	7.56	6.04	*Cbr2*
0.040	Down with MMC	0.35	5.84	4.33	*Cyp2f2*
0.040	Down with MMC	0.35	1.05	− 0.45	*Cdo1*
0.040	Down with MMC	0.36	2.42	0.95	*Sfrp2*
0.039	Down with MMC	0.40	1.28	− 0.05	*Chrna1*

^a^Filtered by the false discovery rate (*q* < *0.05*): unpaired t-test with Benjamini–Hochberg multiple testing correction. Ave Filtration: normalized average expression value of the day 3 filtration bleb sample. Ave MMC: normalized average expression value of the day 3 filtration bleb sample treated with MMC.

### Quantitative-PCR (q-PCR) confirmed reproducibility of the expression analysis

**Figure 2:**
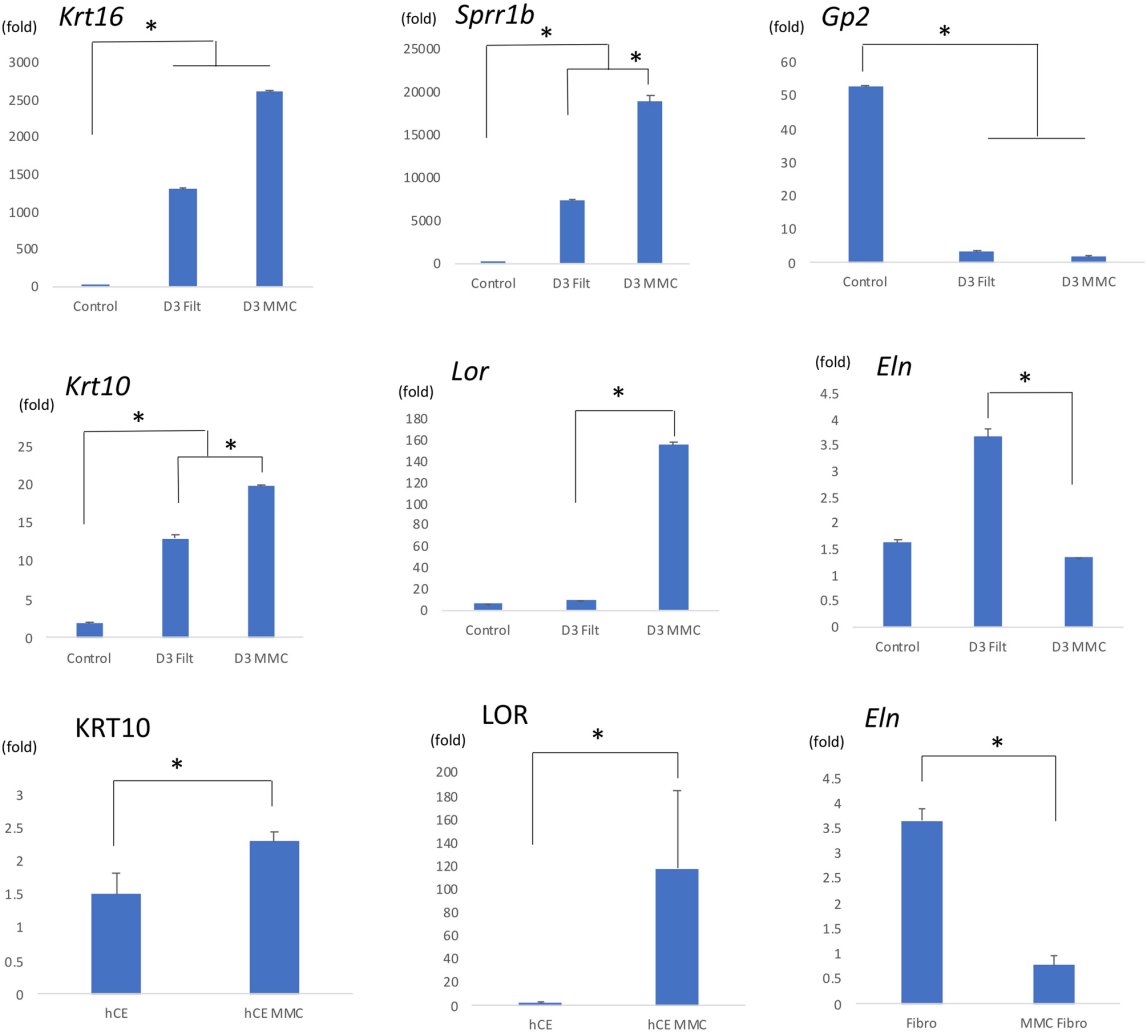
q-PCR analysis of the selected genes. q-PCR analysis was carried out to validate microarray data using replicate cDNA samples. Significant upregulation of *Il6*,* Sprr1b* and downregulation of *Gp2* gene expression were observed in both day 3 samples compared to the control. MMC treatment induced *Krt1*,* Krt10*, and *Lor* gene expression in bleb tissue and abolished filtration-induced *Eln* expression. MMC treatment suppressed *Eln* mRNA expression in mouse conjunctival fibroblasts and KRT1 and LOR mRNA expression in human conjunctival epithelial cells. Error bars indicate means ± s.d. (*P < 0.05, Welch’s *t*-test).

To confirm the reproducibility of the expression array analysis, we carried out q-PCR analyses for selected genes using sets of cDNA samples made in independent experiments. Hyperexpression of the *Krt16* and *Sprr1b* genes and hypoexpression of the *Gp2* gene in a day3 filtration sample compared to a control sample was confirmed (Fig. 2, top row). Hyperexpression of the *Krt10* (pair gene of *Krt1*), and *Lor* genes, and hypoexpression of the *Eln* gene in MMC-treated bleb samples compared to non-MMC-treated bleb samples was found (Fig. 2, middle row). The effects of MMC treatments were further analysed using mouse conjunctival fibroblasts and human conjunctival epithelial (hCE) cells. Upregulation of human KRT10, and LOR gene expression in MMC-treated hCE cells and downregulation of *Eln* gene expression in MMC-treated conjunctival fibroblasts compared to mock-treated control cells were observed (Fig. 2, bottom row).

### Immunohistochemical analysis showed goblet cell depletion in bleb tissue and hyperexpression of keratinization markers in MMC-treated bleb tissue

**Figure 3:**
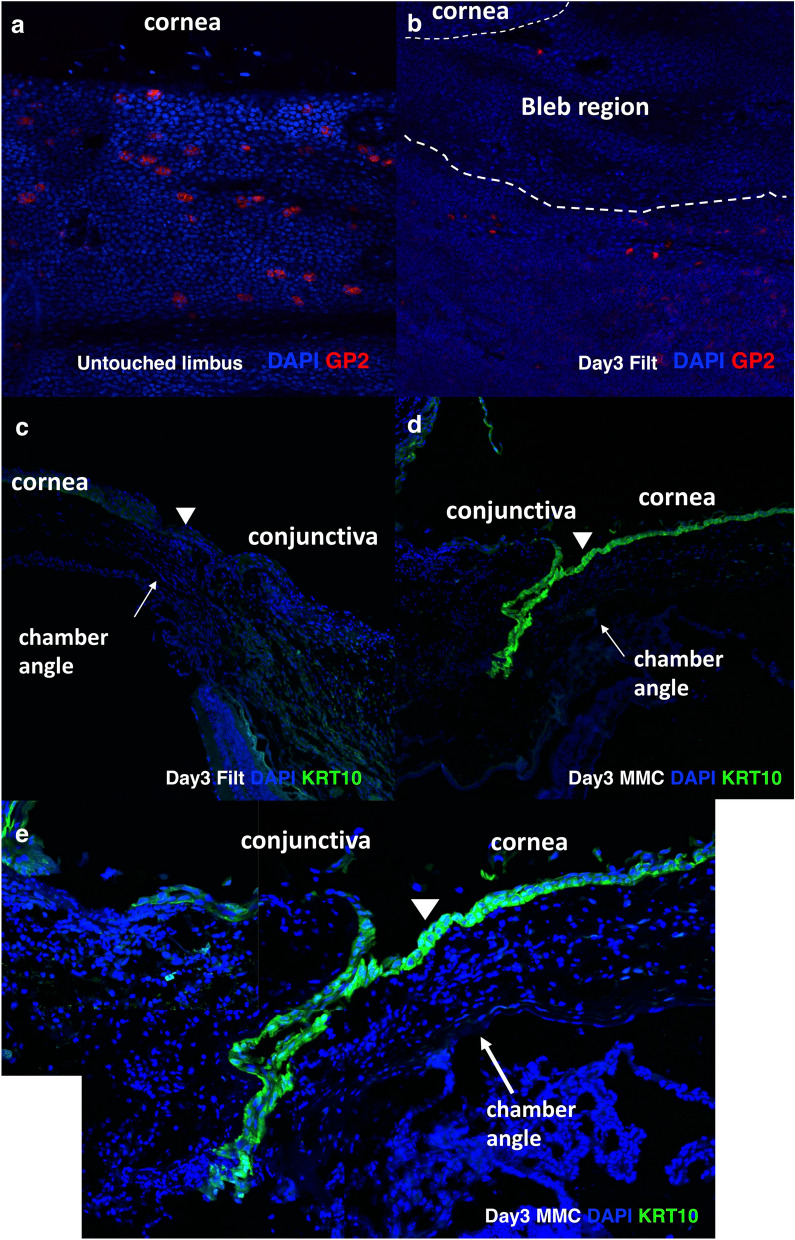
Immunohistochmemical analysis of bleb tissue. Whole mount immunohistochemical analysis of control limbal conjunctiva (**a**) and the bleb region conjunctiva 3 days after surgery (**b**) was carried out using an anti-GP2 antibody. GP2-positive goblet cells were observed in the limbal conjunctiva of the control sample (**a**), whereas no GP2-positive goblet cells were observed in the bleb region (**b**). Nuclear counter staining was carried out with DAPI. Original magnification (200x). Frozen sections of bleb tissue from 3 days after surgery were immunostained with an anti-KRT10 antibody. KRT10-positive epithelial cells (green) is observed in the bleb with MMC treatment (**d**), but no definite KRT-10 staining is observed in the bleb without MMC treatment (**c**). The chamber angles are shown by arrows, and the junctions between corneal and conjunctival epithelia are indicated by arrowheads. A high magnification image of the MMC-treated bleb region shows clear cytoplasmic KRT10-positive cells in the conjunctival and corneal epithelia of the MMC-treated bleb (**e**). Nuclear counter staining was carried out with DAPI. Original magnification: 100 × for c and d; 200 × for e.

We compared GP2 protein expression in the naïve limbal tissue and bleb region (3 days after filtration surgery) by whole mount immunohistochemical staining. GP2-positive goblet cells were observed in the naïve limbal conjunctiva (Fig. 3a), but no GP2-positive staining was observed in the bleb region (Fig. 3b). Using frozen sections of the day 3 bleb, KRT10-immunopositive signals were observed at the conjunctival epithelial cells of the bleb region with MMC treatment (Fig. 3d,e), but no KRT10-staining was observed at the conjunctival epithelium in the bleb without MMC treatment (Fig. 3c).

## Discussion

This is the first study of transcriptome analysis using a mouse model of filtration surgery. Our results showed that the needle tips were patent at least until day 3 (Fig. 1) and we selected day 3 as the timepoint to investigate the early phase transcriptome profiles of filtration surgery. Several groups reported mouse filtration surgery models using a 25G- or 30G-needles to make a fistula in the limbal region of the C57BL/6 mouse^10–12^. In a preliminary experiment we also tried to create a filtration models by making fistulae using 30G needles and found that the results were more variable for the fistula model. Our model is much more consistent for forming a bleb, at least at day 3 (Fig. 1b). Our mouse filtration surgery model is clinically relevant because we have been using an Ex-Press Mini Glaucoma Shunt (Alcon, Fort Worth, TX) made of stainless steel in our clinic.

In preliminary experiments, we also made samples from mock-treated eyes, in which conjunctival dissections and two limbal 11-0 nylon sutures were done without needle tip insertion into the anterior chamber. The gene expression profiles of mock-treated tissue were more similar to the operated bleb than to the control (untouched) conjunctiva, and there were only 27 hyperexpressed (increase of expression in MMC-bleb) and 18 hypoexpressed (decrease of expression in MMC-bleb) DEGs (fold change > 2, FDR *q* < 0.05) in comparison between mock samples and MMC-treated bleb samples (Supplementary Table S3), and no DEGs were found in comparison of the mock and bleb samples without MMC treatment. We think that the wound healing process of filtration surgery was the sum of ocular surface wound healing and the effect of aqueous humor filtration, so we decided to use naive conjunctival tissue as control tissue to analyze the gene expression profiles of filtration surgery.

For microarray analysis, we took great care to sample the bleb region reproducibly. We used disposable biopsy punches 2 mm in diameter, which made it possible to obtain samples from the bleb region with the same size. We also took care to avoid contamination by the lens, retinal and iris tissues (Supplementary Fig. 3).

To evaluate the hyperexpressed transcripts in filtration samples, we sorted the results by the order of fold changes and obtained a list of the top 39 hyperexpressed transcripts (Table 1). The list included *Sprr1b* (small proline-rich protein 1B), which is a known marker for keratinization in dry eye conditions^13^. Small proline-rich proteins (SPRRs) are cross-linked to themselves or to other cornified envelope proteins like loricrin (LOR) and keratin, and play roles in keratinization^14^. Through gene ontology (GO) pathway analysis, we found enrichment of keratinization-related gene expression in the hyperexpressed transcripts (Supplementary Table S6). These results suggested keratinization to be one of the features of early alteration of gene expression in filtration surgery.

When we compared our list of 755 hyperexpressed transcripts (Supplementary Table S1) with the previous rat filtration surgery experiment data obtained from day 2 and day 5 after surgery^5^, we found that some of the wound healing-related transcripts (*Fabp5*, *Fn1*,* Mmp3*,* Mmp9*,* Timp1*), and remodeling-associated transcripts (*Col1a1*,* Col3a1*) were hyperexpressed in common. This observation suggested that activation of wound healing/remodeling-related transcripts was a common feature of filtration surgery, as also suggested by review articles^15,16^. Yu-Wai-Man et al. reported transcriptome analysis of conjunctival fibroblasts after human glaucoma surgery^17^. They compared fibroblasts established from fibrotic and non-fibrotic bleb tissues, and found increased expression of *MYOCD*,* LMO3*,* IL-6*,* RELB* and decreased expression of *PRG4*,* CD34*,* IL-33*,* MMP-10*,* WISP2*,* COL6A6*,* IGFBP5* in fibroblasts established from fibrotic bleb tissue. Consistent with their results, our results showed an increase of *IL6* in the bleb region at day 3 (Table 1).

We defined a relatively high cutoff value for the expression level for the control conjunctival tissue (> 3 for normalized expression value) to elucidate meaningful hypoexpressed genes in the filtration samples (Table 2). We considered that hypoexpressed transcripts relevant to pathophysiology should be homeostatic ones, in which relatively abundant expression should be observed in control conjunctiva. In fact, we found *Gp2* (a molecular marker for goblet cells), *Adh1* (alcohol dehydrogenase 1)^18^ and *Gsta3* (glutathione S-teransferase)^19^, which are known as epithelial cell homeostasis-related genes, among the top 21 hypoexpressed transcripts. GO pathway analysis also showed the glutathione metabolic process as a pathway enriched in the hypoexpressed gene set (Supplementary Table S6). These results suggested that the expression of homeostatis-related genes was compromised by the injury of filtration surgery.

Using sets of cDNAs obtained via independent experiments, we confirmed some of the biologically meaningful DEGs from our microarray results by q-PCR analysis. The result confirmed upregulation of *Krt16* (damage-induced, hyperproliferative keratin)^20^, and *Sprr1b*,* and* downregulation of *Gp2* in day 3 filtration samples, consistent with the microarray results (Fig. 2, top row).

MMC treatment is widely used in human trabeculectomy surgery. It augments the filtration effect and contributes to the success rate of trabeculectomy^9^. On the other hand, MMC treatment has negative effects; for examples, it induces transconjunctival oozing and leaks, and it is associated with blebitis^21^. We found that MMC treatment induced expression of keratinization-related genes, including a set of late cornified envelope (*Lce1d*,* Lce1e*,* Lce1f*), *Lor*, and *Krt1*genes (Table 3). Our results are consistent with a previous histological report of MMC-treated bleb tissue showing the keratinization of conjunctival epithelium^22^.

We further analyzed the effects of MMC treatment in vitro. Mouse conjunctival fibroblasts were treated with MMC and a decrease of *Eln* gene expression was confirmed (Fig. 2, bottom row). MMC treatment of human conjunctival epithelial (hCE) cells induced keratinization-related human KRT10 and LOR mRNA expression (Fig. 2, bottom row). These results showed that MMC-treatment affected multiple types of conjunctival tissues (fibroblasts and epithelial cells).

Immunohistochemical analysis of the bleb tissue showed depletion of GP2-positive staining in the bleb region of day 3 filtration samples (Fig. 3b). Agnifili et al. reported decreased goblet cell density associated with failures of filtration surgery^23^. Amar et al. found reduction of MUC5AC staining in MMC-treated blebs using an impression cytology technique, suggesting the loss of proper goblet cell functions in the MMC-treated bleb tissue^24^. Thus, we considered it important to prevent downregulation of epithelial cell homeostasis-related genes, including *Gp2*, after filtration surgery because a healthy ocular surface is important for successful filtration surgery.

We also found KRT10 protein expression (Fig. 3d,e) and LOR protein expression (Supplementary Fig. S5) in the conjunctival epithelium of the MMC-treated bleb but not in the bleb without MMC treatment. We used the KRT10 antibody instead of KRT1 because of the good quality of the antibody for immunohistochemical staining. KRT10 is a keratin molecule paired with KRT1 and their keratin expression is associated with epithelial keratinization^25^. Using q-PCR analysis, we confirmed that hyperexpression of mouse *Krt10* and human KRT10 genes was induced by MMC treatment (Fig. 2). KRT10 expression is associated with ocular surface diseases like Stevens Johnson syndrome and superior limbic keratoconjunctivitis, and KRT1/KRT10 are not expressed in healthy conjunctival tissue^26,27^. Another study reported that ectopic KRT10 expression in mucosal tissue was associated with morphological changes and inflammatory cell infiltration^28^. We are now planning further studies to investigate the relations between ocular surface keratinisation and bleb status by impression cytology of human bleb tissue.

**Figure 4:**
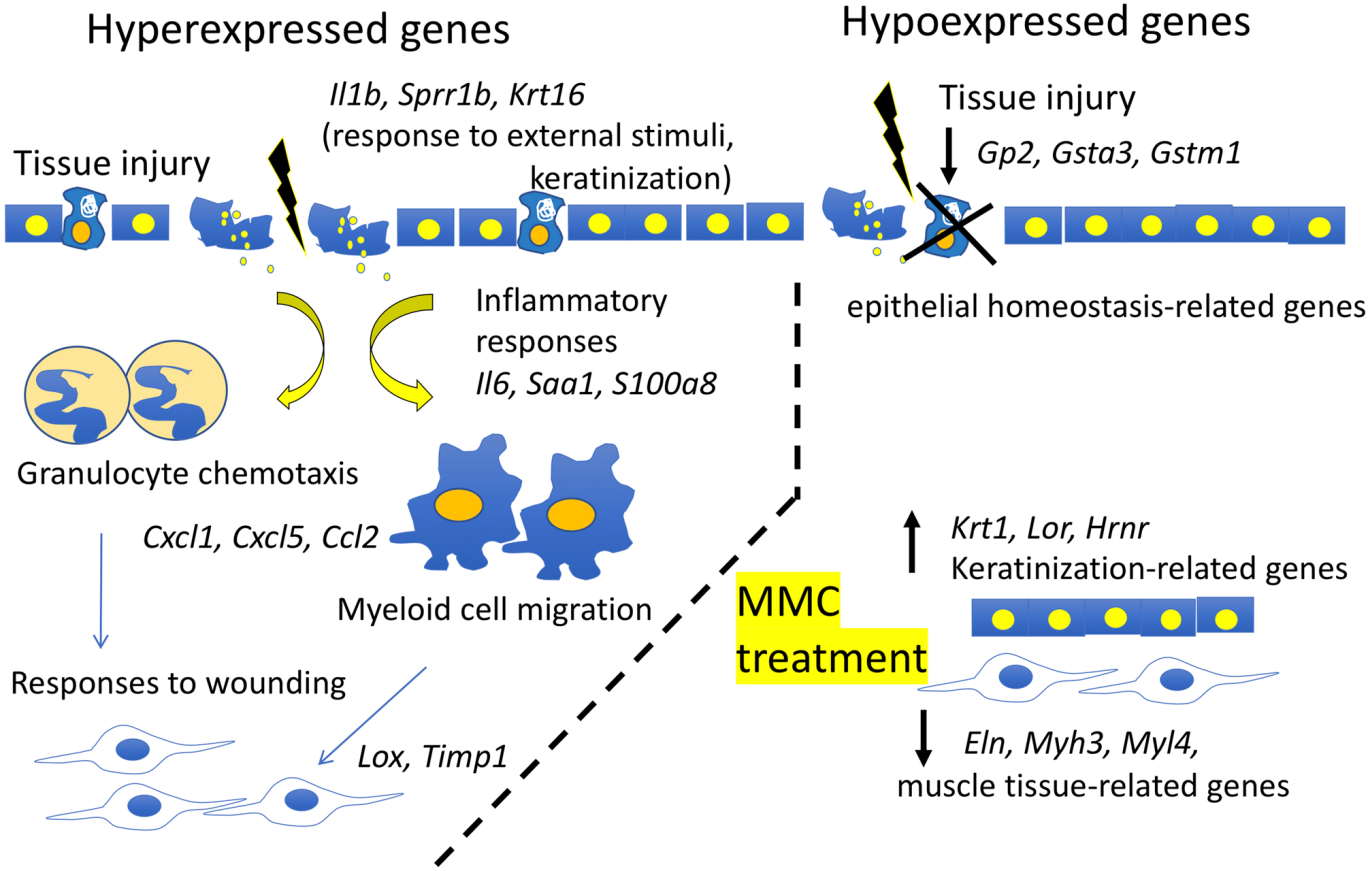
Schematic summary of microarray analysis. We found hyperexpression of keratinization-related genes (*Krt16*,* Sprr1b*) and of gene expression (*Il1b*) in response to external stimuli (tissue injury). Hyperexpression of inflammatory response-related genes (*Il6*,* Saa1*,* S100a8)* and genes related to the chemotaxis of inflammatory cells (*Cxcl1*,* Cxcl5*,* Ccl2*) was also observed. On the other hand, hypoexpression of the goblet cell marker (*Gp2*) suggests breakdown of epithelial homeostasis. We also found upregulation of keratinization-related transcripts (*Krt1*,* Lor*,* Hrnr*) and downregulation of muscle tissue-related transcripts (*Eln*,* Myh3*,* Myl4*) after MMC treatment of the bleb region.

We summarized our microarray results in Fig. 4 in consideration of the results of GO pathway analysis (Supplementary Table S6). Hyperexpression of epithelial keratinization-related genes (*Krt16* and *Sprr1b*) is a previously unrecognized finding. The increases of inflammatory response-related genes (*Il1b*,* Il6*), inflammatory cell chemotaxis-related genes (*Cxcl1*,* Cxcl5*,* Ccl2*), and wound healing-related genes (*Lox*,* Timp1*) are consistent with previous reports^15,16^. Genes related to conjunctival epithelial cell homeostasis and keratinization status (*Gp2*,* Krt1/Krt10*,* Lor*) will be possible targets for further improvement of the outcome of filtration surgery. For example, it was reported that dry eye treatments could suppress epithelial cell metaplasia and loss of goblet cells in the ocular surface^29^.

In conclusion, we determined transcriptome profiles using a mouse filtration surgery model. By combining gene-modified mouse and/or cell depletion mouse models, this mouse filtration surgery model will be a useful tool for elucidating the roles of particular genes or cells during filtration.

## Methods

### Mouse filtration surgery model

All of the procedures of animal experiments were approved by the animal experiment research committee of Juntendo University (No. 1324) and adhered to the ARVO Statement on the Use of Animals in Ophthalmic and Vision Research (https://www.arvo.org/About_ARVO/Policies/Statement_for_the_Use_of_Animals_in_Ophthalmic_and_Visual_Research/). Using BALB/c mice, a filtration surgery model was made by incision of the limbal conjunctiva, followed by the insertion of a 33G needle tip into the anterior chamber, and 11-0 nylon sutures were placed on the limbus (Supplementary Fig. S1). For some groups of mice, 5 μl of 0.4 mg/ml MMC was injected under the resected conjunctiva by micropipette, and the excess amount of MMC solution was wiped off 3 min after injection.

### Microarray analysis

We used 18 mice for microarray analysis. Only the left eyes of the mice were used for filtration surgery. The right eyes were used as controls. Independent microarray analysis was carried out three times. For one analysis, the filtration bleb, the filtration bleb with MMC and the control tissue were analyzed. One pooled-RNA sample was made up from three eye tissues either extracted from three bleb tissues or three control conjunctival tissues (Supplementary Fig. S4). The operated eyes were enucleated and preserved in RNA*later* (Thermo Fisher Scientific Japan, Tokyo, Japna) after euthanasia. The bleb tissue was isolated, using a 2 mm biopsy punch (Kai, Tokyo, Japan) and homogenized using an ultrasound homogenizer. For control samples, the same part of the contralateral eye was isolated by the same procedures. Total RNA was collected using a NucleoSpin RNA (Macherey–Nagel, Germany), and gene array analysis was carried out using an Agilent 8 × 60 K mouse expression array according to the manufacturer’s protocol. All the RNA samples were evaluated using an Agilent 2,100 bioanalyzer and verified as good quality (RIN > 9.4). Data normalization and analysis were carried out using GeneSpring GX software (Agilent Japan, Tokyo, Japan). We excluded transcripts under the background expression level from both control and filtration model samples. Selection methods for differentially expressed genes (DEGs) are summarized in Supplementary Fig. S4. We defined differentially expressed genes (DEGs) as those with a false discovery rate (FDR) of *q* < *0.05* and a fold change > 4 for hyperexpressed genes. We then sorted the hyperexpressed transcripts by the order of fold changes and selected the top 39 transcripts abundantly expressed in the filtration bleb samples. For the hypoexpressed genes, we selected DEGs among the transcripts abundantly expressed in the control conjunctival tissue (> 3 for normalized expression value) fulfilling the conditions of FDR (*q* < *0.05*) and a fold change < 0.5. Comparing the day 3 filtration bleb samples from mice with and without MMC treatment, the top 21 MMC-induced transcripts (fold change > 25, FDR *q* < 0.05, corrected average expression value of the MMC treated bleb > 1) were determined (Table 3). We also carried out gene ontology (GO) enrichment analysis (https://geneontology.org/docs/go-enrichment-analysis/) to find biological pathways enriched in the lists of DEGs.

### Q-PCR analysis

For quantitative PCR (q-PCR) analysis, we prepared other sets of operated eye samples, and cDNA was prepared using a random primer and the reverse transcriptase (ReverTra Ace; both from Toyobo, Osaka, Japan) according to the manufacturer’s protocol. We used 6 mice for q-PCR analysis (two mice for one analysis). The left eyes of the mice were used for filtration surgery and the right eyes were used as controls. For one analysis, three conditions; (1) the filtration bleb, (2) the filtration bleb with MMC and (3) control tissue, were analyzed. Each cDNA sample was made from one eye tissue either extracted from one bleb tissues or one control conjunctival tissues. Independent q-PCR analysis was carried out three times, and the results were essentially the same. Representative data are shown.

We also prepared cDNA from cultured mouse conjunctival fibroblasts and human conjunctival epithelial cells. Mouse conjunctival fibroblasts were established using conjunctival tissue obtained from wild-type Balb/c mice and used for this study within 5 passages. Those fibroblasts were maintained using DMEM supplemented with 10% fetal calf serum. Subconfluent conjunctival fibroblasts in 12-cell culture plates were incubated with either DMEM containing 0.4 mg/ml of MMC or DMEM only for 3 min and washed three times with PBS. hTERT-immortalized human conjunctival epithelial cells (hCE cells: a generous gift from Dr. Satoshi Kawasaki, Osaka University) were maintained as previously described using SHEM medium^30^. Subconfluent hCE cells were treated with either 0.4 mg/ml of MMC in DMEM or DMEM only for 3 min, and washed three times with PBS. After changing to fresh SHEM medium, the fibroblasts and hCE cells were further cultured for another 48 h and cDNAs were prepared for each condition. The-above mentioned in vitro experiments were repeated three times, and a representative data are shown. All the q-PCR analysis was carried out using a q-PCR System (Light Cycler 96 System, Roche Molecular Systems, Tokyo, Japan) with KAPA SYBR Fast qPCR mixture (KAPA Biosystems, Cape Town, South Africa). The expression of differentially expressed genes (*Gapdh*, *Krt16*,* Sprr1b*,* Gp2*,* Eln*,* Krt10*, GAPDH, KRT10, ELN) was quantified by q-PCR. The relative gene expression was quantified by comparative Ct methods using *Gapdh* (for mouse) and GAPDH (for human) expression in the same cDNA as the controls. The primer pairs used in this study are summarized in Supplementary Table S4.

### Statistical analysis

Statistical evaluations of microarray analysis were performed using the unpaired t-test. The results were filtered by the false discovery rate (FDR: *q* < 0.05) using Benjamini–Hochberg multiple testing correction (Supplementary Fig. S4). Statistical evaluations of q-PCR analysis were performed using the two-tailed unpaired Welch’s t-test. *P* < 0.05 was considered statistically significant.

### Immunohistochemistry

The bleb tissue was sampled at 3 days after surgery and analyzed by immunohistochemical staining. We used 6 mice for immunohistochemical analysis, utilizing frozen sections, and the left eyes of the mice were used for filtration surgery. Two conditions: (1) three eyes for filtration blebs and (2) three eyes for filtration blebs with MMC, were analyzed. We use another 3 mice for whole mount immunohistochemistry. Two conditions: (1) three left eyes for filtration blebs and (2) three right eyes as a control, were analyzed. Immunohistochemical analysis was carried out essentially as previously described^31^. In brief, eyes with the bleb region were immediately fixed with 4% paraformaldehyde (PFA) in PBS for 3 h. For GP2 immunohistochemical staining, whole mount immunohistochemical staining^32^ was carried out using conjunctival tissue permeabilized with 0.01% Triton-X 100 in PBS for 5 min. A rat anti-mouse GP2 monoclonal antibody (MBL, Nagoya Japan) was used as the primary antibody and a donkey Alexa 594-conjugated anti-rat IgG antibody (Life Technology Japan, Tokyo, Japan) was used as the secondary antibody. Three eyes with blebs and their three contralateral eyes were used for whole mount staining. For KRT10 and loricrin (LOR) immunostaining, the bleb samples were immersed with 30% sucrose in PBS, and frozen in Optimal Cutting Temperature Compound (Sakura Finetek, Tokyo, Japan) using liquid nitrogen. Five-micrometer frozen sections were made and air-dried. A rabbit anti-mouse KRT10 polyclonal antibody and a rabbit anti-mouse LOR antibody (BioLegend, San Diego, CA) were used as the primary antibodies. Isotype-matched control antibodies were used instead of the primary antibodies as negative controls. A donkey Alexa 488-conjugated anti-rabbit IgG antibody (Life Technology Japan) was used as the secondary antibody. The samples were visualized using a Fluoview 1000 confocal laser microscope (Olympus, Tokyo, Japan).

## Supplementary information

Supplementary Information.

Supplementary Tables.
