# Supplementary material for: Alteration of gene expression in mice after glaucoma filtration surgery
Source: Sci Rep. 2020 Sep 14;10:15036. doi: 10.1038/s41598-020-72036-0 (PMC7490364; doi:10.1038/s41598-020-72036-0)
Supplement: Supplementary file 1 — Supplementary Information. [file 41598_2020_72036_MOESM1_ESM.pdf]

Alteration of gene expression in mice after glaucoma filtration surgery

Keisuke Adachi, Yosuke Asada, Toshiaki Hirakata, Miki Onoue, Satoshi Iwamoto, Toshimitsu Kasuga and Akira Matsuda.

Supplementary Fig. S1

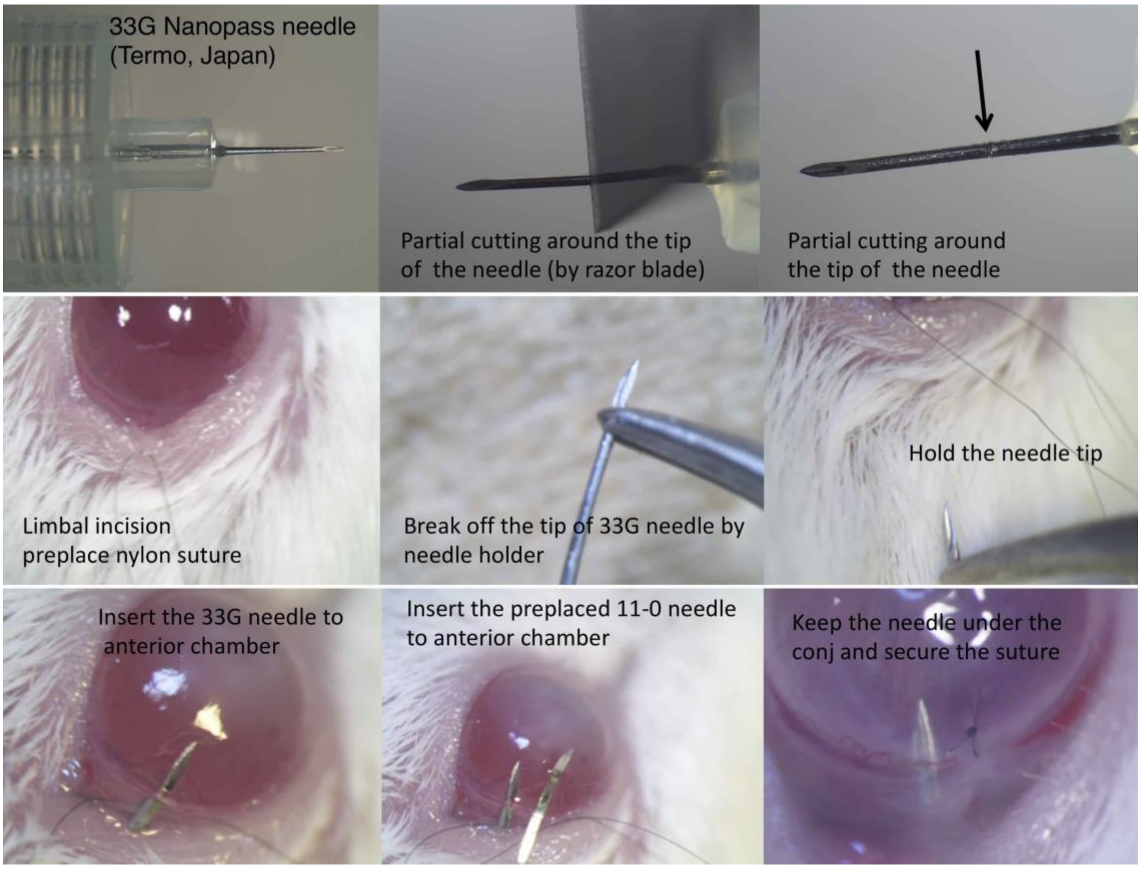

Supplementary Fig. S1. Preparation of the needle tip and surgical procedures of mouse filtration surgery model

Partial cutting around the tip of a 33G Nanopass needle (Termo, Japan) was done with a disposable razor blade (upper line). The mouse filtration surgery model was made by

incision of the limbal conjunctiva, followed by the insertion of the 33G needle tip into the anterior chamber, and the root of the inserted needle tip was covered by the conjunctiva using one or two 11-0 nylon sutures secured to the limbus (middle and lower lines).

Supplementary Fig. S2

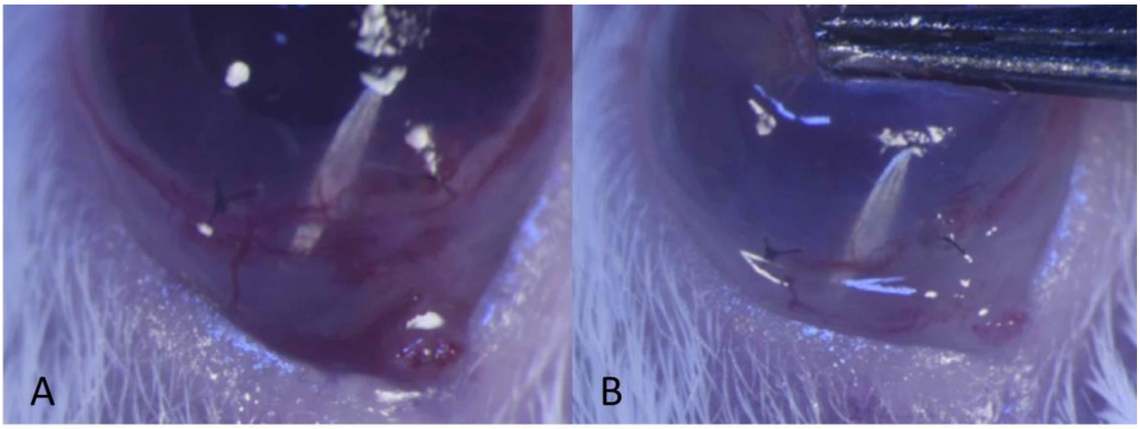

Supplementary Fig. S2. Reformation of bleb by pressure on the cornea on day 7

Flat bleb region at 7 days after filtration surgery (a). With gentle pressure on the cornea, the bleb was reformed (b).

Supplementary Fig. S3

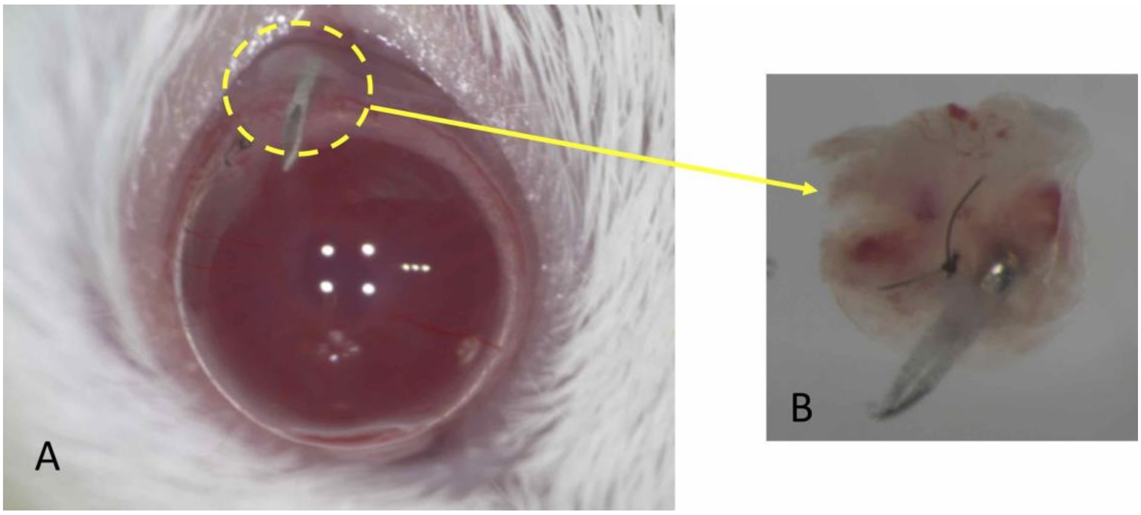

Supplementary Fig. S3. Sampling of the bleb region with biopsy punches

The bleb region (a) was trepanised with a 2 mm biopsy punch (indicated by the dotted yellow circle), and the bleb region was sampled (b) for microarray and q-PCR analyses.

Supplementary Fig. S4

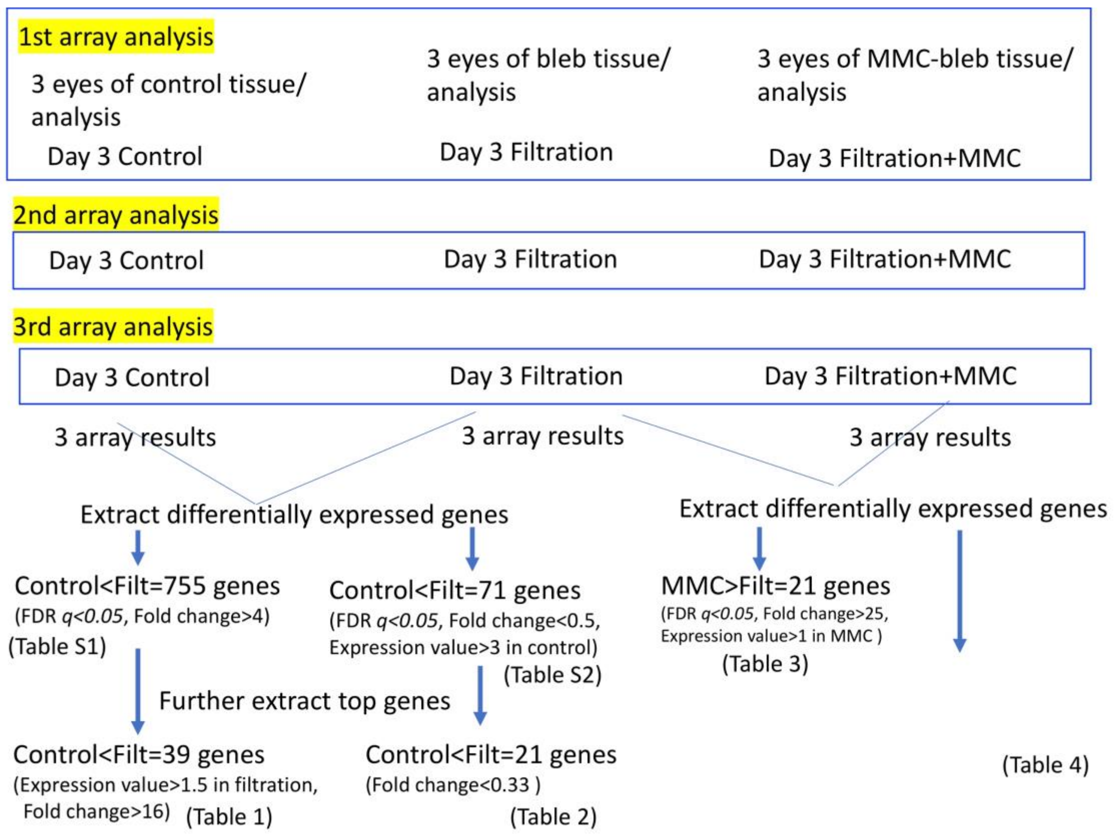

Supplementary Fig. S4 Procedures of microarray analyses and subsequent data selections

A schematic of the microarray analyses and procedures and subsequent data selections is shown.

Supplementary Fig. S5

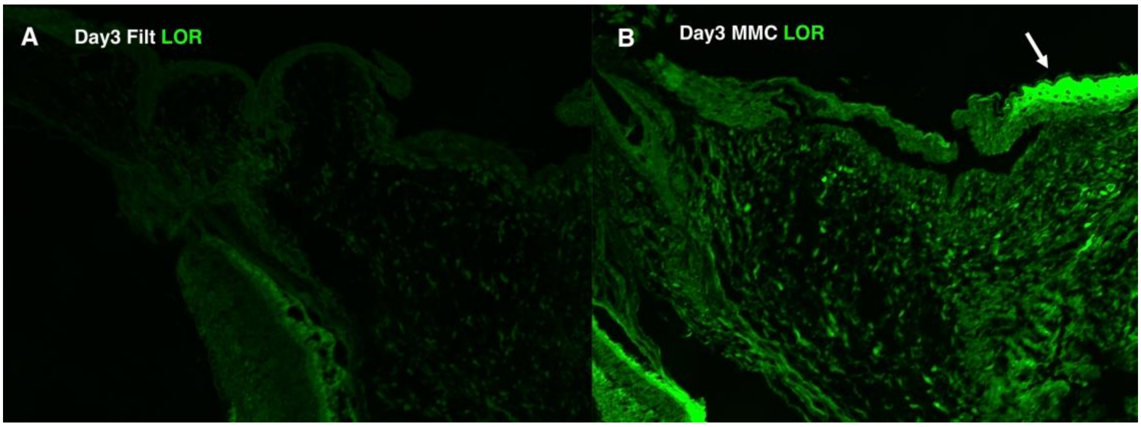

Supplementary Fig. S5. Immunohistochemical staining of the bleb tissue using an anti-LOR antibody

Immunohistochemical analysis of bleb tissue from 3 days after filtration surgery without MMC (a) and with MMC treatment (b) was carried out using an anti-LOR antibody. LOR positive conjunctival epithelium is observed at the MMC treated bleb (arrow), but not in the bleb region without MMC treatment. Original magnification (100x).

Table legends for supplementary Tables

Table S1. 755 hyperexpressed transcripts (filtration>control)

*Filtered by the false discovery rate ($q<0.05$): unpaired t-test with Benjamini-Hochberg

multiple testing correction. Ave Filtration: normalized average expression value of the day 3 filtration bleb sample. Ave control: normalized average expression value of the control sample.

Table S2. 71 hypoexpressed transcripts (filtration<control)

*Filtered by the false discovery rate ($q<0.05$): unpaired t-test with Benjamini-Hochberg multiple testing correction. Ave Filtration: normalized average expression value of the day 3 filtration bleb sample. Ave control: normalized average expression value of the control sample.

Table S3. Differentially expressed genes in comparison between MMC-treated bleb tissue and mock-treated conjunctiva

*Filtered by the false discovery rate ($q<0.05$): unpaired t-test with Benjamini-Hochberg multiple testing correction. Mock Ave: normalized average expression value of the day 3 mock-treated bleb region tissue. MMC Ave: normalized average expression value of the day 3 filtration bleb sample treated with MMC.

Table S4. Primer pairs used in this study

Table S5. Gene ontology (GO) analysis of the top 39 hyperexpressed transcripts

GO analysis was carried out using the top 39 hyperexpressed transcripts (Table 1). Among the 124 enriched pathways, we selected 6 representative GO pathways (asterisks) considering the redundancies of the pathways.

Table S6. Summaries of the gene ontology (GO) analysis of Tables 1, 2, 3, 4

GO analysis was carried out using the DEGs listed in Tables 1 to 4. Uploaded genes in each GO pathway are also shown.
